# Hippocampal CA2 Lewy pathology is associated with cholinergic degeneration in Parkinson’s disease with cognitive decline

**DOI:** 10.1186/s40478-019-0717-3

**Published:** 2019-04-25

**Authors:** Alan King Lun Liu, Tsz Wing Chau, Ernest Junwei Lim, Idil Ahmed, Raymond Chuen-Chung Chang, Michail E. Kalaitzakis, Manuel B. Graeber, Steve M. Gentleman, Ronald K. B. Pearce

**Affiliations:** 1 Neuropathology Unit, Division of Brain Sciences, Department of Medicine, Imperial College London, 4/F, Burlington Danes Building, Du Cane Road, London, W12 0NN UK; 2 Laboratory of Neurodegenerative Diseases, School of Biomedical Sciences, LKS Faculty of Medicine, The University of Hong Kong, Pokfulam, Hong Kong SAR; 3 State Key Laboratory of Brain and Cognitive Sciences, The University of Hong Kong, Pokfulam, Hong Kong, Special Administrative Region of China; 4 Brain and Mind Centre, Bosch Institute, Discipline of Anatomy and Embryology, and Charles Perkins Centre, Faculty of Medicine and Health, The University of Sydney, 94 Mallett Street, Camperdown, NSW 2050 Australia

**Keywords:** Parkinson’s disease, Alzheimer’s disease, Cholinergic system, Diagonal band of Broca, CA2, Hippocampus

## Abstract

Although the precise neuropathological substrates of cognitive decline in Parkinson’s disease (PD) remain elusive, it has long been regarded that pathology in the CA2 hippocampal subfield is characteristic of Lewy body dementias, including dementia in PD (PDD). Early non-human primate tracer studies demonstrated connections from the nucleus of the vertical limb of the diagonal band of Broca (nvlDBB, Ch2) to the hippocampus. However, the relationship between Lewy pathology of the CA2 subfield and cholinergic fibres has not been explored. Therefore, in this study, we investigated the burden of pathology in the CA2 subsector of PD cases with varying degrees of cognitive impairment and correlated this with the extent of septohippocampal cholinergic deficit. Hippocampal sections from 67 PD, 34 PD with mild cognitive impairment and 96 PDD cases were immunostained for tau and alpha-synuclein, and the respective pathology burden was assessed semi-quantitatively. In a subset of cases, the degree of CA2 cholinergic depletion was quantified using confocal microscopy and correlated with cholinergic neuronal loss in Ch2. We found that only cases with dementia have a significantly greater Lewy pathology, whereas cholinergic fibre depletion was evident in cases with mild cognitive impairment and this was significantly correlated with loss of cholinergic neurons in Ch2. In addition, multiple antigen immunofluorescence demonstrated colocalisation between cholinergic fibres and alpha-synuclein but not tau pathology. Such specific Lewy pathology targeting the cholinergic system within the CA2 subfield may contribute to the unique memory retrieval deficit seen in patients with Lewy body disorders, as distinct from the memory storage deficit seen in Alzheimer’s disease.

## Introduction

The clinical presentation of Parkinson’s disease (PD) displays a high degree of heterogeneity, not limited to the classical quartet of motor signs [50]. It has been increasingly recognised that non-motor symptoms produce a significant impact on the quality of life of both patients and their carers [15, 23, 26, 48]. In particular, cognitive impairment is prevalent among PD patients and progression to dementia is common in the later stages of disease [37]. Clinically, PDD patients present with more severe attention deficits and impairment in executive function and visuospatial function as compared with Alzheimer’s disease (AD), with relative sparing of language functions [27]. In terms of the memory domain, retrieval memory is more affected in PDD whereas encoding and storage memory decline in AD [27]. As a result, the Movement Disorder Society Task Force established consensus criteria for the diagnosis of PDD [27] and PD with mild cognitive impairment (PD-MCI) [53] to distinguish these entities from other types of cognitive impairment.

Despite extensive clinicopathological studies conducted over the last few decades, the exact neuropathological substrate contributing to cognitive decline in PD remains unclear. The neuropathology of PDD is similar to dementia with Lewy bodies (DLB) [52], which is thought to fall within the same spectrum of disease as Lewy body dementia (LBD). AD-type pathology including hyperphosphorylated tau and amyloid-beta (Aβ) is common in LBD, but its burden is often not as severe as cases with pure AD [9, 34, 45, 66]. The burden of alpha-synuclein (αSN) pathology in the form of cortical Lewy bodies (LB) was found to be associated with the severity of cognitive decline in PD cases, even in the absence of AD pathology [49, 56]. However, recent studies have shown that a multifactorial combination of LB and AD pathology burden may be a better predictor of dementia in PD [18, 39, 40]. Although motor impairment in PD is correlated with the depletion of dopamine, there is limited improvement of cognitive function in PD patients on dopamine replacement therapy [32, 38], suggesting that the development of PDD is probably due to other neurochemical deficits. The “cholinergic hypothesis” proposed in the 1970s and 80s emphasised the importance of acetylcholine in cognition [10]. Initially focused primarily on AD brains, cholinergic dysfunction in PD and LBD was found to be at least as severe as that seen in AD patients, as evidenced from studies on neuronal loss in the acetylcholine-producing cells of the nucleus basalis of Meynert (nbM) [63].

There has been recent interest in the hippocampal CA2 subfield following better morphological and physiological characterisation of the hippocampal subregions [24, 62]. It was previously reported that ubiquitin-positive dystrophic neurites were present in the CA2/3 subregions of DLB cases which differentiates them from AD [21, 22]. These were later found to be αSN-immunopositive Lewy neurites which frequently co-exist with cortical Lewy bodies [44] and were significantly associated with the presence of dementia in PD [47]. In a more recent study, Hall and colleagues found Lewy neuritic pathology in the CA2 subregion even in non-demented PD cases. It was also found that the increased αSN pathology in the CA2 region was associated with a reduction of cholinergic activity, as measured with a choline acetyltransferase (ChAT) assay [33]. Coincidentally, the CA2 subfield was found to have the highest density of ChAT-positive fibres [64] with early non-human primate tracer studies identifying the origin of cholinergic hippocampal innervation from the medial septal nucleus (MSN; Ch1) and the nucleus of the vertical limb of the diagonal band of Broca (nvlDBB, Ch2) [59]. Although no significant changes were found between PD and PDD cases [33], that particular study was underpowered due to its limited sample size. Furthermore, significant depletion of cholinergic neurons in the Ch1 and Ch2 was found in DLB cases compared with AD cases [30].

Based on evidence from existing studies, we hypothesise that cholinergic dysfunction contributes to progressive cognitive decline in PD and is associated with increased Lewy pathology within the CA2 hippocampal subfield. We aimed to study tau and αSN pathology burden in the CA2 subsector in PD cases with different degrees of cognitive impairment. We also investigated the degree of cholinergic degeneration in the CA2 subsector and the Ch2 to identify septohippocampal cholinergic pathway involvement. Finally, we explored associations between tau, αSN and cholinergic processes in the CA2 subfield.

## Methods

### Cases

Post-mortem human brain samples used in this study were provided by the Parkinson’s UK Tissue Bank at Imperial College London (Registered charity in England and Wales (258197) and in Scotland (SC037554)). Tissue sections containing the hippocampus and the nvlDBB were obtained.

The diagnosis of PD was based on established clinical and neuropathological criteria [20, 43]. In addition, Braak αSN stage and modified Braak tau stage were assigned based on the recommended assessment protocol outlined by BrainNet Europe (BNE) [3, 4]. Briefly, Braak αSN stage was assigned based on the topographical distribution of αSN-immunoreactive inclusions in the medulla, pons, midbrain, basal forebrain, hippocampus, and cingulate, temporal, frontal and parietal cortical regions. Braak tau stage was assessed using 4 sections immunostained for tau, including the visual cortex including the calcarine fissure, the middle temporal gyrus, the anterior hippocampus and the posterior hippocampus. Retrospective case-note analysis was performed by a movement disorder specialist (RKBP) and research postgraduate (AKLL). The cognitive status of patients was obtained from their clinical records. PD patients with cognitive deficits severe enough to interfere with independent activities of daily living, satisfying DSM-IV [5] and ICD-10 [67] clinical criteria for dementia and Movement Disorder Society Task Force diagnostic criteria for PDD [27] were classified as PDD. PD patients with significant cognitive deficits in any cognitive domain including memory, executive function, visuospatial function, attention and language, typically accompanied by psychotic symptoms with visual hallucinations, but not to the extent that they prevented independent activities of daily living, were classified as PD with mild cognitive impairment (PD-MCI).

### Selection and exclusion criteria

Only cases with available hippocampal sections with the CA2 region clearly visible, adequate tissue fixation and good tissue quality were selected. Cases were excluded if the clinical notes were incomplete or of poor quality. Hence, only cases with clinical follow-up within 24 months before death were included. Cases with extensive vascular lesions in the brain including cerebral infarcts, haemorrhage, severe CAA pathologies and small vessel disease, which may contribute to cognitive decline, were excluded. Cases with severe co-existing AD pathology (Braak tau staging IV or above) were also excluded. Finally, cases with co-existing neuropathology including tumours, demyelinating lesions, fronto-temporal lobar dementia, amyotrophic lateral sclerosis, Creutzfeldt-Jakob disease and other Parkinsonism pathologies including progressive supranuclear palsy (PSP), corticobasal degeneration (CBD) and multiple systems atrophy (MSA), were also excluded.

### Semi-quantitative analysis of hippocampal CA2 pathology

**Fig. 1:**
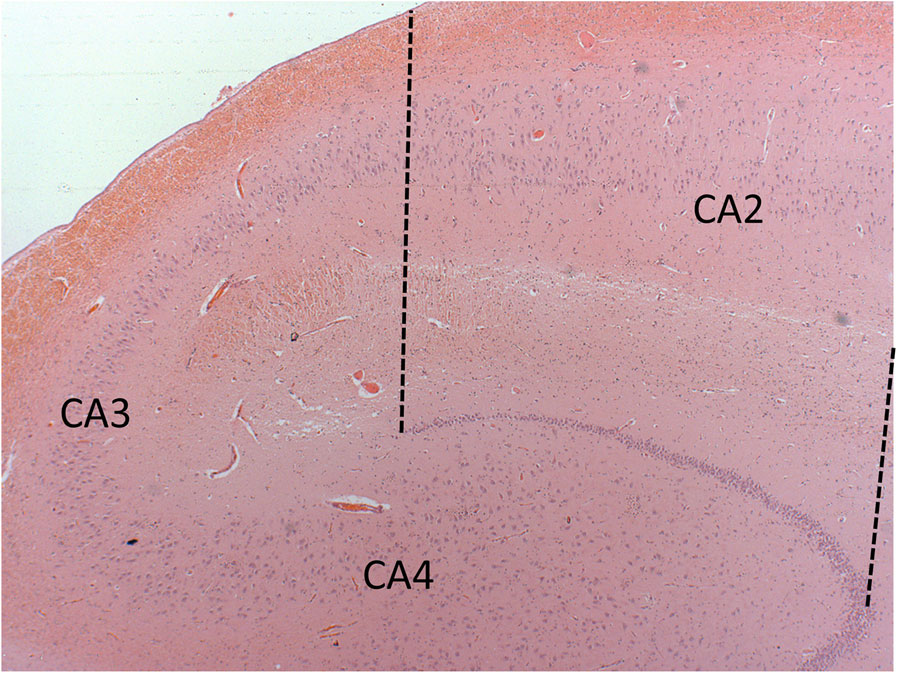
Photomicrograph illustrating the hippocampal CA2 subfield. The CA2 region was defined as the pyramidal cell layer parallel to the boundaries set by the ends of the granule cells of the external limbs of the dentate gyrus

**Fig. 2:**
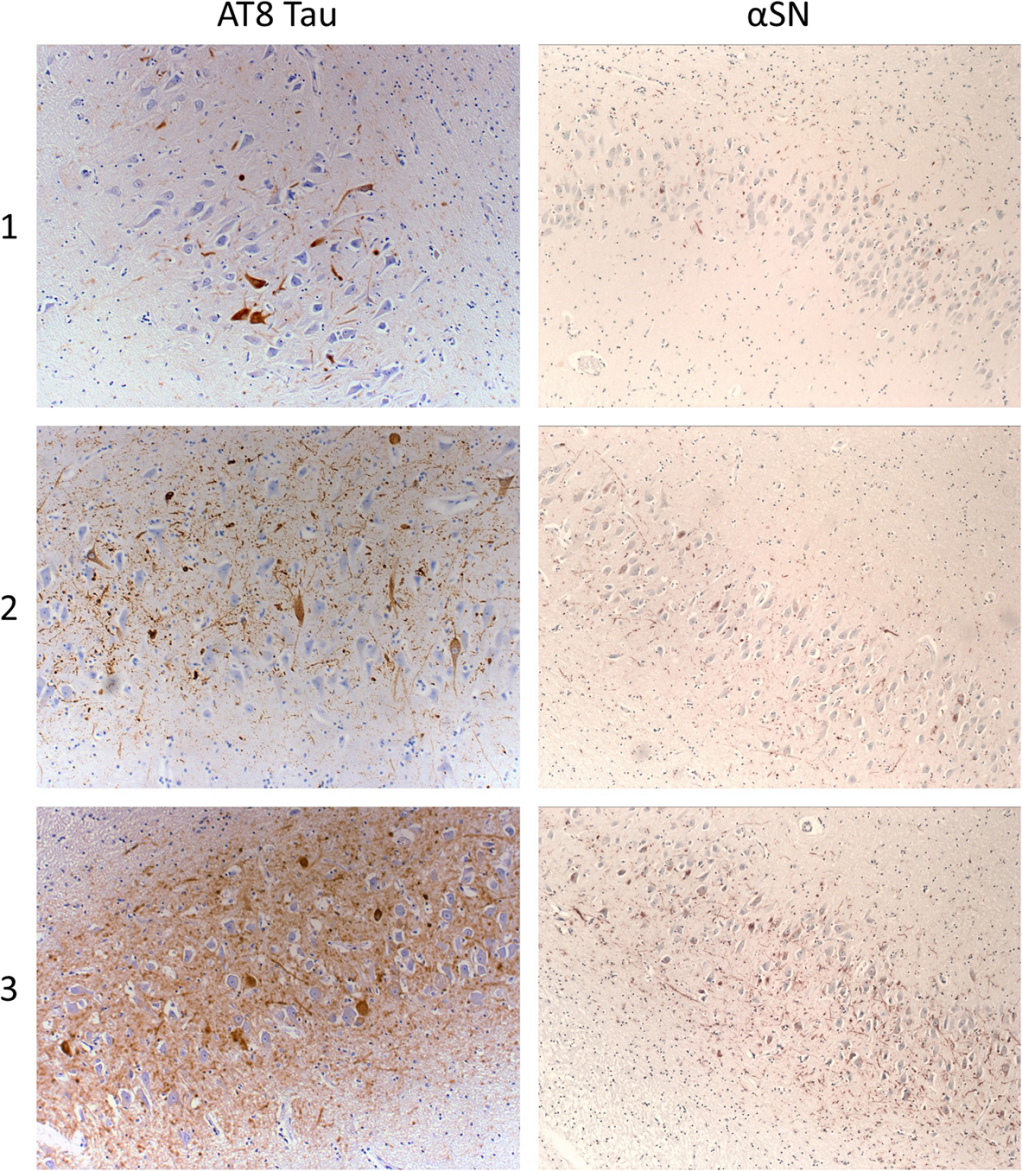
Representative photomicrographs of semi-quantitative assessment of tau and αSN pathology. Neuritic tau and αSN burden was graded from 0 (absence to pathology) to 3 (abundant pathology) using immunostaining against AT8 tau (phospho-PHF-tau pSer202 + Thr205 tau epitope) and αSN. Figures illustrates grading from 1 to 3. Magnification 200x for tau and 100x for αSN

For the first part of the study, formalin-fixed, paraffin-embedded diagnostic slides (7 μm thick) containing the CA2 region of the hippocampus, stained for αSN and tau (Phospho-PHF-tau pSer202 + Thr205) were obtained from the tissue bank. Serial haematoxylin and eosin (H&E) stained slides were used as a guide for the identification of the CA2 region. We defined the CA2 region as the pyramidal cell layer parallel to the boundaries set by the ends of the granule cells of the external limbs of the dentate gyrus (Fig. 1). This delineation method has been applied previously in the literature [6, 12]. Semi-quantitative analysis was performed by three investigators (SMG, AKLL and TWC) blinded to the clinical diagnosis of the cases. Immunostained hippocampal sections were initially screened under a × 10 stage objective and assessment of pathology was then carried out under a × 20 stage objective. Overall Lewy pathology burden and neuritic tau burden were graded from 0 (absence to pathology) to 3 (abundant pathology) (Fig. 2). In total, 3–4 sections per case were analysed in this study.

### Choline acetyltransferase immunohistochemistry and immunofluorescence

Cholinergic neurons in the nvlDBB (Ch2) and cholinergic varicosities in the hippocampal CA2 region were visualised using immunohistochemistry and immunofluorescence respectively for choline acetyltransferase (ChAT). For this study, tissue sections at a coronal level just anterior to the anterior commissure decussation were obtained for Ch2 quantification as the highest density of cholinergic neurons is found at this level (as Fig. 4l in [54]). Tissue sections were dewaxed in two changes of xylene (10 min, 5 min) and rehydrated through decreasing concentration of industrial methylated spirit (IMS)/ethanol (100, 100, 90, 70%; 5 min each) and subsequently in distilled water (5 min). For immunohistochemistry, basal forebrain sections (containing the nvlDBB) were immersed in 1% hydrogen peroxide (H_2_O_2_)/phosphate buffered saline (PBS, pH 7.4) for 30 min at room temperature for quenching of endogenous peroxidase activity. Next, tissue sections were pretreated with autoclaving (25 min) in 0.01 M sodium citrate buffer (pH 6) before rinsing in PBS (3 × 5 min) and incubation with anti-ChAT antibodies (1:100 diluted with 0.3% Triton-X 100 in PBS; AB144P; Millipore, UK) at 4 °C overnight. 2% rabbit serum (for basal forebrain sections) or 2% donkey serum (for hippocampal sections) was added in the antibody diluent for blocking of non-specific staining.

On the second day, tissue sections were washed with PBS (3 × 5 min) and signal amplification techniques were carried out. In the case of standard immunohistochemistry, sections were incubated with biotinylated rabbit-anti-goat secondary antibodies (1:100 diluted in 0.3% Triton-X 100 in PBS; BA-5000; Vector Laboratories, UK) for 1 h at room temperature. After rinsing with PBS (3 × 5 min), sections were incubated with avidin-biotin complex using the VECTASTAIN Elite ABC Kit (PK-6100; Vector Laboratories, UK) for 1 h at room temperature. Subsequently, tissues were washed in PBS (3 × 5 min) and visualised with 3′3-diaminobenzidine (DAB) (5 min). Tissue sections were then washed in distilled water (2 × 5 min) and briefly counterstained with haematoxylin. Finally, tissues were rehydrated and cleared through an increasing concentration of IMS/ethanol (70, 90, 100, 100%; 5 min each) and in xylene (2 × 5 min), before coverslipping with Distrene-Plasticiser-Xylene (DPX).

For immunofluorescence, hippocampal sections were incubated with Alexa Fluor 488-conjugated donkey anti-goat secondary antibody (1:200; A-11055; ThermoFisher Scientific, UK) for 1 h at room temperature. Sections were then rinsed briefly in PBS (3 × 5 min) and incubated for 10 min in 0.3% Sudan black B dissolved in 70% ethanol to block endogenous autofluorescence by lipofuscin, before coverslipping and mounting with VECTASHEILD antifade mounting medium with DAPI (Vector Laboratories, UK).

### Multiple-antigen immunofluorescence for choline acetyltransferase, alpha-synuclein and tau

Double and triple immunofluorescence were used to investigate the relationship between αSN and/or tau pathology and cholinergic fibres in the hippocampus. Sections were dewaxed and rehydrated as described in the section above. For double staining with anti-ChAT and anti-AT8 tau antibodies, tissues were pretreated with autoclaving (25 min) in 0.01 M sodium citrate buffer (pH 6). Additional incubation in 80% formic acid (10 min) was needed for double staining with anti-ChAT and anti-αSN antibodies. Tissue sections were then incubated with various antibodies combinations overnight (Table 1). The remaining protocol follows as described in the section above.

**Table 1:** Antibodies used for immunohistochemistry and multiple-antigen immunofluorescence staining in this study

Antibody	Host	Clonality	Immunogen	Company	Catalogue number	Dilution	Pretreatment	Secondary antibody
Choline-acetyltransferase (ChAT)	Goat	Polyclonal	Human placental ChAT	Millipore	AB144P	1:100 (IHC); 1: 50 (IF)	Pressure cooker, 0.01 M sodium citrate buffer (pH 6)	Alexa-Fluor® 488-conjugated donkey anti-goat antibody
Tau (AT8; Phospho-PHF-tau pSer202 + Thr205)	Mouse	Monoclonal (IgG_1_)	Partially purified human PHF-Tau	Pierce Thermo Scientific	MN1020	1:100 (IF)	Nil	Alexa-Fluor® 568-conjugated or Alexa-Fluor® 647-conjugated donkey anti-mouse antibody
Alpha-synuclein (Clone 42)	Mouse	Monoclonal (IgG_1_)/ Clone 42	Rat Synuclein-1 aa. 15–123	BD Transduction Laboratories	610,787	1:100 (IF)	10 mins 80% formic acid	Alexa-Fluor® 647-conjugated donkey anti-mouse antibody
Tau (ps396; phosph-tau pSer396)	Rabbit	Polyclonal	Human Tau, serine 396	Thermo Fisher Scientific	44752G	1:200 (IF)	Nil	Alexa-Fluor® 568-conjugated donkey anti-rabbit antibody

Abbreviations: IF, immunofluorescence; IHC, immunohistochemistry

Triple Immunofluorescence was conducted for ps396-tau, αSN and ChAT with the same protocol as the double immunofluorescence as described above. However, a sequential staining approach was applied where anti-ps396 tau primary and secondary antibodies were applied on the 2nd and 3rd days after the incubation of anti-ChAT and anti-αSN primary and secondary antibodies. Pretreatment with autoclave was conducted before formic acid for optimal immunoreactivity [46].

### Confocal imaging

Single and multiple immunofluorescent-stained slides were visualised and imaged using a Zeiss LSM-780 inverted confocal microscope (Carl Zeiss, Germany) at the Facility for Imaging by Light Microscopy (FILM) at Imperial College London. × 10 objectives (Plan-Apochromat Ph1 M27; numerical aperture, 0.45; working distance, 2.0 mm), × 63 objective (Plan-Apochromat, oil immersion; DIC M27; numerical aperture, 1.40; working distance, 0.19 mm) with laser excitation at 405 nm (Diode), 488 nm (Argon multiline), 543 nm (HeNe) and 594 nm (HeNe) were used. Image capture and processing were performed using the Zen Black (Carl Zeiss, Germany) software.

### Quantification of cholinergic varicosities in the CA2 hippocampal subfield

**Fig. 3:**
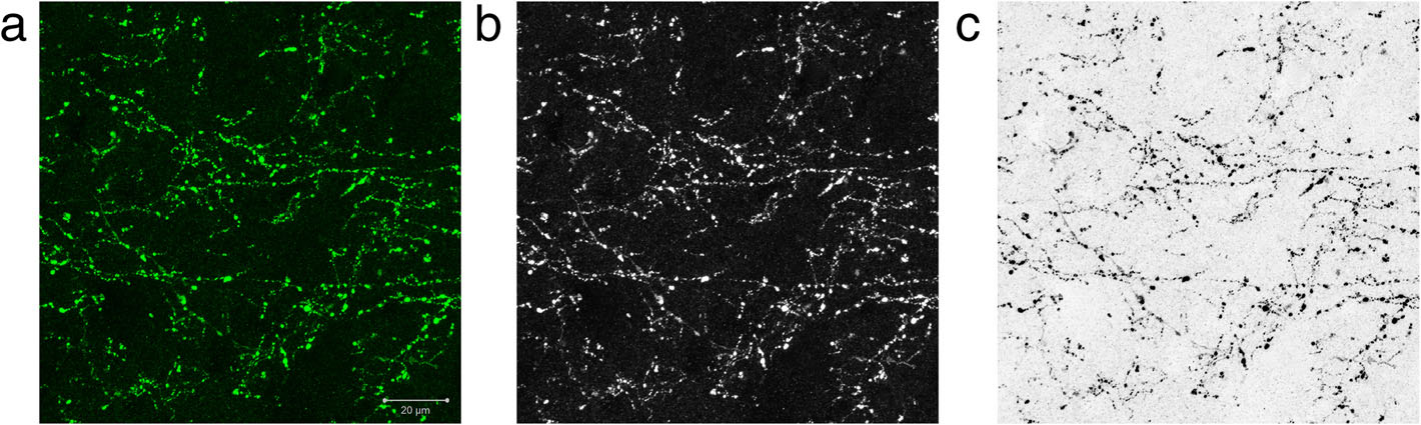
Quantification of cholinergic varicosities in the CA2 hippocampal subfield. Acquired imaged from the confocal microscope (**a**) was converted to monochrome by extraction of the green channel (**b**). The image was then colour-inverted (**c**) and subsequently, the percentage area coverage of ChAT was obtained with manual thresholding on the ImagePro Plus software. Scale bar = 20 μm

Stained hippocampal sections were screened using a × 10 stage objectives to locate the CA2 hippocampal subfield and quantitative assessment of ChAT-immunopositive fibres was carried out under a × 63 stage objective, blinded to the clinical diagnosis, at the region of maximal ChAT immunoreactivity to ensure consistency of the assessment. The full thickness of the section was imaged with z-stacking and the maximal intensity projection was acquired post-acquisition using the Zen Black software. The image was exported and analysed using the ImagePro Plus (Media Cybernetics, Inc., USA) image analysis software as described in Fig. 3. A size exclusion parameter was used (Area; range 10–150) to exclude dystrophic Lewy neurites which were also immunopositive for ChAT. The investigator was blinded to the diagnosis of the case throughout the whole data acquisition process.

### Quantification of cholinergic neurons in the nucleus of the vertical limb of diagonal band of Broca

ChAT-immunostained sections were visualised and images captured with a light microscope (Olympus AHBT3 VANOX) with digital camera at × 4 stage objective. Basal forebrain cholinergic cell groups often consist of a compact and diffuse sector [35]. For this reason, the area of maximal ChAT-immunopositive cell density was captured using the ImagePro Plus software. Next, the blue channel was extracted from the image with contrast enhancement. By manually adjusting the threshold of intensity and size with the captured image on the side, the number of immunopositive neurons was counted. Any cells touching the border of the image were not included in the quantification. Overlapping cells were manually separated with a ‘separate cluster’ function on the program, and non-specific staining including tissue folds and vascular structures was manually ‘toggled-off’.

### Statistical analysis

Statistical analysis was carried out and graphs were generated using Statistical Package for the Social Sciences (SPSS v25, IBM) and GraphPad Prism software (Version 7.02). Data in this study was not normally distributed, hence Kruskal-Wallis and Mann-Whitney U tests were used for comparisons of ChAT-positive neurons and varicosities, and tau and Lewy pathology burden among the cases. Post-hoc pairwise analysis with Dunn’s correction was used for multiple comparisons. Spearman’s rho was used for correlation between ChAT-positive neuron count in the nvlDBB and ChAT-positive varicosities in the CA2 hippocampal region. Statistical significance is reached when *p* < 0.05.

## Results

### Case demographics

In summary, 197 cases comprised of 67 PD, 34 PD-MCI and 96 PDD cases were selected for this study. All cases were matched in age at death, disease duration, and Braak αSN and tau stages (Table 2). There was no significant difference in the post-mortem interval between different cohorts (range 24.7–26.6 h).

**Table 2:** Patients demographics for CA2 αSN and tau burden study

Diagnosis	PD	PD-MCI	PDD
*n*	67	34	96
M:F (% M)	40:27 (59.7%)	20:14 (58.8%)	68:28 (70.8%)
Mean age at onset (SD)	65.06 (8.75)	62.91 (11.97)	64.81 (10.49)
Mean age at death (SD)	77.34 (6.92)	76.85 (8.32)	78.03 (7.39)
Mean duration of disease (SD)	12.36 (6.38)	14.09 (6.52)	13.28 (6.44)
Mean post-mortem interval (hours)	24.67	26.56	25.15
Median Braak αSN stage	6	6	6
Median Braak tau stage	2	2	2

### Burden of Lewy and tau pathology in the hippocampal CA2 subfield in PD cases

**Fig. 4:**
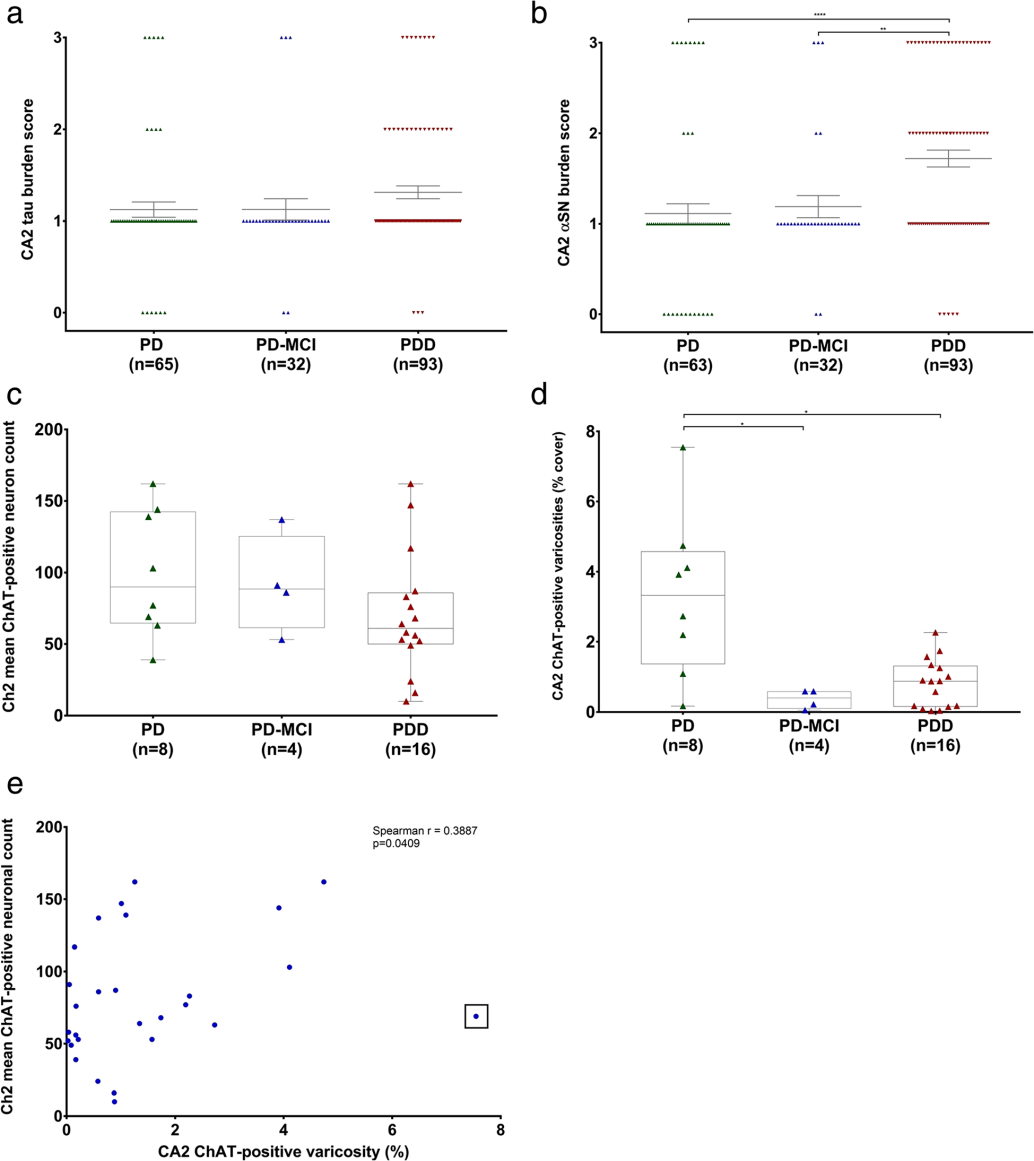
CA2 hippocampal subfield proteinaceous pathology burden and septohippocampal cholinergic deficit in PD with varying degrees of cognitive deficit. Scatter plots comparing tau (**a**) and αSN (**b**) pathology in PD, PD-MCI and PDD. Note that 7 diagnostic slides for tau analysis and 9 for αSN analysis had to be excluded due to poor tissue quality and the difficulty in accurately identifying the CA2 subfield. Error bars showing mean ± SEM. Box-and-whiskers plots comparing nvlDBB (Ch2) mean cholinergic neuron count (**c**) and CA2 ChAT-positive varicosities (**d**) between PD, PD-MCI and PDD cases. Correlation scatter graph showing the relationship between Ch2 mean cholinergic neuronal count and CA2 hippocampal subfield ChAT-positive varicosity (**e**). An outlier was observed and is indicated by a box. *p < 0.05; **p < 0.01; ****p < 0.0001

Semi-quantitative assessment of Lewy and tau pathologies were performed on diagnostic hippocampal sections. However, 7 diagnostic slides for tau analysis and 9 for αSN analysis had to be excluded due to poor tissue quality and the difficulty in accurately identifying the CA2 subfield. There was no difference in CA2 tau burden among PD cases with differing degrees of cognitive deficit, although a trend towards a higher burden in demented cases could be seen (Fig. 4a). In terms of αSN pathology, PDD cases had a significantly higher αSN burden compared with PD-MCI (*p* = 0.0074) and PD cases without cognitive deficits (*p* < 0.0001) (Fig. 4b). It was interesting to note that 169 cases (89.9%) had at least some αSN pathology in the hippocampal CA2 sector and among those without any known cognitive deficit, only 12 (19.0%) had absence of any Lewy pathology. Correlation analysis revealed no significant correlations between duration of disease and hippocampal CA2 tau (Spearman rho = − 0.059; 2-tailed *p* = 0.421) or αSN (Spearman rho = 0.135; 2-tailed *p* = 0.065) pathology.

### Anterior basal forebrain and hippocampal cholinergic depletion in Parkinson’s disease

Cases with tissue sections containing the nvlDBB at the coronal level just anterior to the anterior commissure decussation were obtained and immunostained for ChAT for the quantification of cholinergic neurons. Since the nvlDBB was not a routinely sampled area at the tissue bank, only 8 PD, 4 PD-MCI and 16 PDD cases with representative anterior basal forebrain section and suitable hippocampal sections were available for this part of the study (Table 3). Cases were matched for age, disease duration and pathological staging as above. Although no significant difference in mean ChAT-positive neuronal count was detected, a trend for a step-wise decrease in neuronal density could be seen with increasing cognitive deficit (Fig. 4c).

**Table 3:** Patients demographics for Ch2 cholinergic neuronal count and hippocampal CA2 cholinergic varicosity quantification

Diagnosis	PD	PD-MCI	PDD
*n*	8	4	16
M:F (% M)	4:4 (50%)	3:1 (75%)	13:3 (81.3%)
Mean age at onset (SD)	62.13 (8.68)	62.25 (13.72)	65.31 (12.40)
Mean age at death (SD)	73.50 (5.90)	81.00 (8.04)	78.19 (5.97)
Mean duration of disease (SD)	11.38 (5.71)	19.00 (5.72)	12.81 (8.30)
Mean post-mortem interval (hours)	31.00	14.00	27.63
Median Braak αSN stage	6	6	6
Median Braak tau stage	1	2	2

**Fig. 5:**
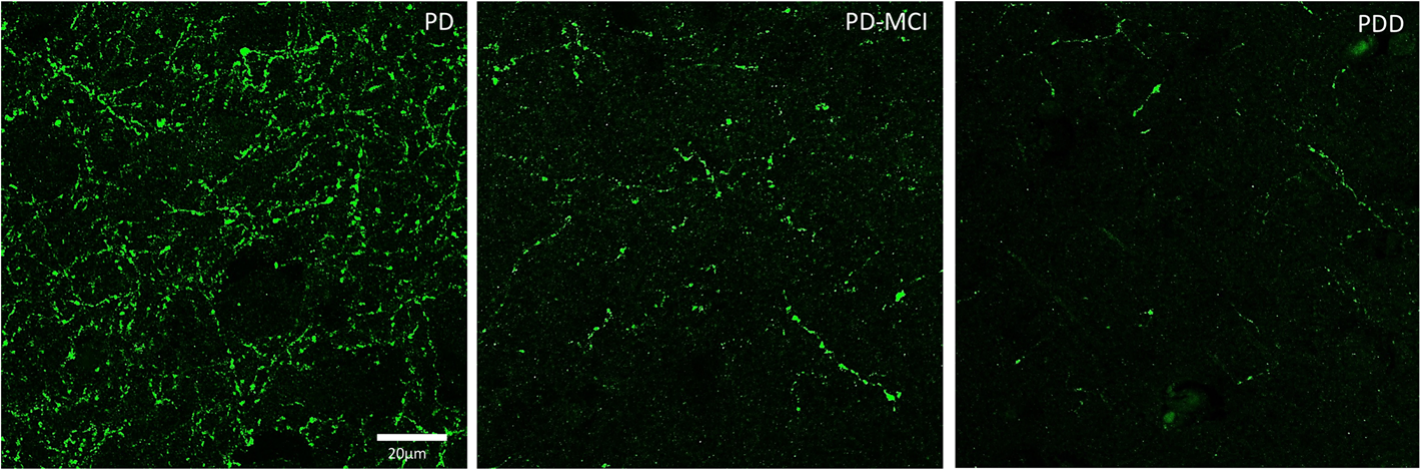
Representative photomicrographs of ChAT terminal immunofluorescence in the hippocampal CA2 in PD, PD-MCI and PDD cases. Images acquired using confocal microscope at ×63 magnification. Scale bar = 20 μm

Hippocampal sections of the selected cases were also immunostained for ChAT for the quantification of cholinergic varicosities within the CA2 subfield. A significant decrease in ChAT-positive varicosities was found in PD-MCI (*p* = 0.0275) and PDD (*p* = 0.0207) cases compared with PD cases with no cognitive impairment (Fig. 4d, Fig. 5). However, it was important to note that there was a large variability in PD cases without cognitive deficits. There was a small but significant correlation between changes in ChAT-positive varicosities in the CA2 subfield and changes in cholinergic neuronal density within the Ch2 (Spearman’s rho = 0.3887; two-tailed *p* = 0.0409) (Fig. 4e). However, we identified an outlier in ChAT-immunopositive varicosity density (box, Fig. 4e) as it falls over 2.5 standard deviations from the mean value (1.465%). With the outlier excluded, a moderate correlation was seen (Spearman’s rho = 0.4183; two-tailed *p* = 0.0299). No correlation between ChAT-positive varicosities in the CA2 and αSN burden (Spearman’s rho = − 0.09; *p* = 0.664) or tau burden (Spearman’s rho = 0.004; *p* = 0.986) was found.

### Colocalisation of Lewy pathology and cholinergic varicosities in the CA2 hippocampal subfield

**Fig. 6:**
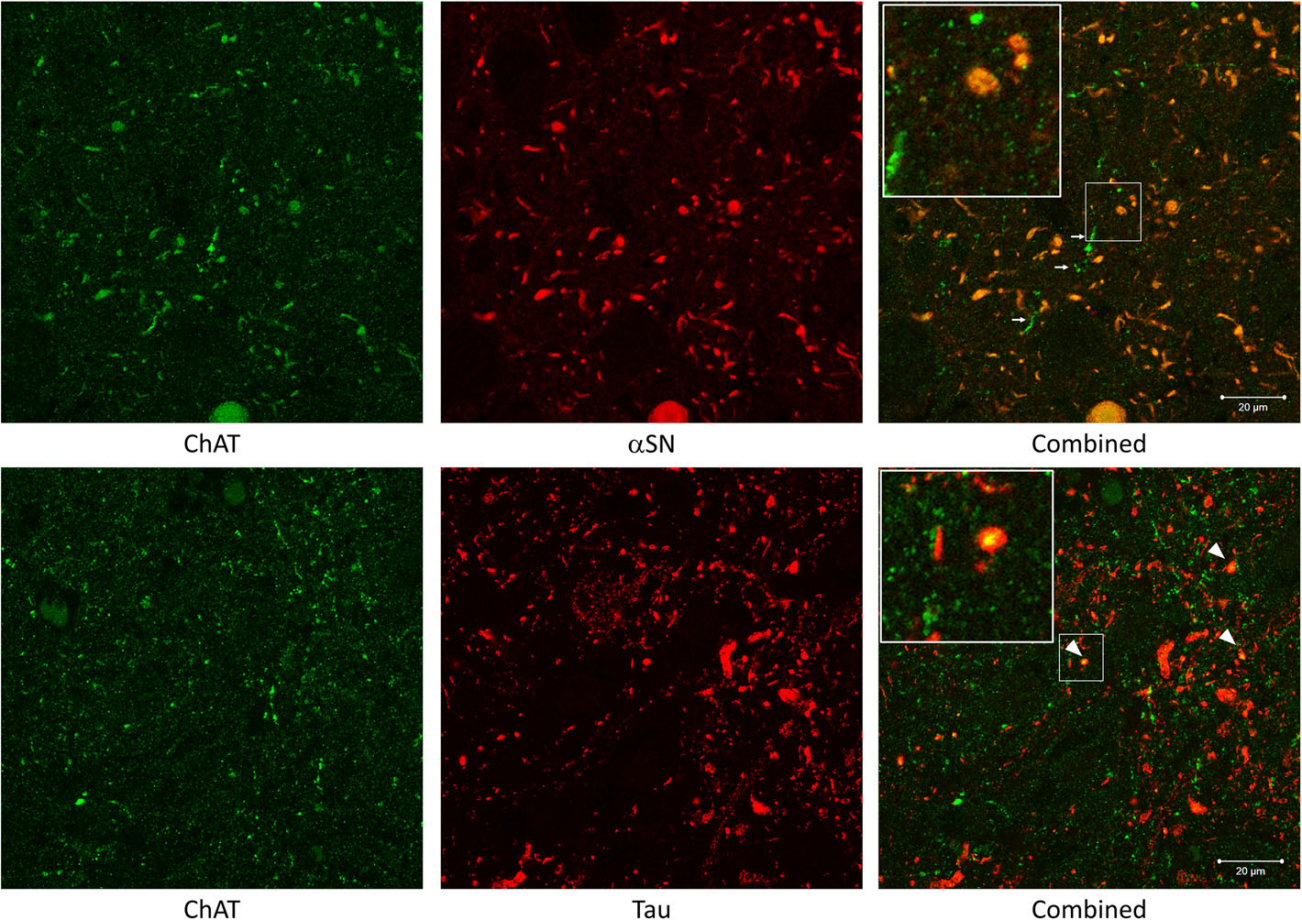
Double immunofluorescence staining between ChAT and αSN or tau. Post-acquisition image processing was performed to enhance the contrast of the images. On the top, double immunofluorescence staining with ChAT (green) and αSN (red) showed near complete co-localisation (yellow). Some ChAT-positive neuronal fibres had sparing of αSN pathology (white arrows). At the bottom, double immunofluorescence staining with ChAT (green) and AT8 tau (red) only had minimal colocalisation (white arrowheads). Insets showing co-localisation in higher magnification. Scale bars = 20 μm

**Fig. 7:**
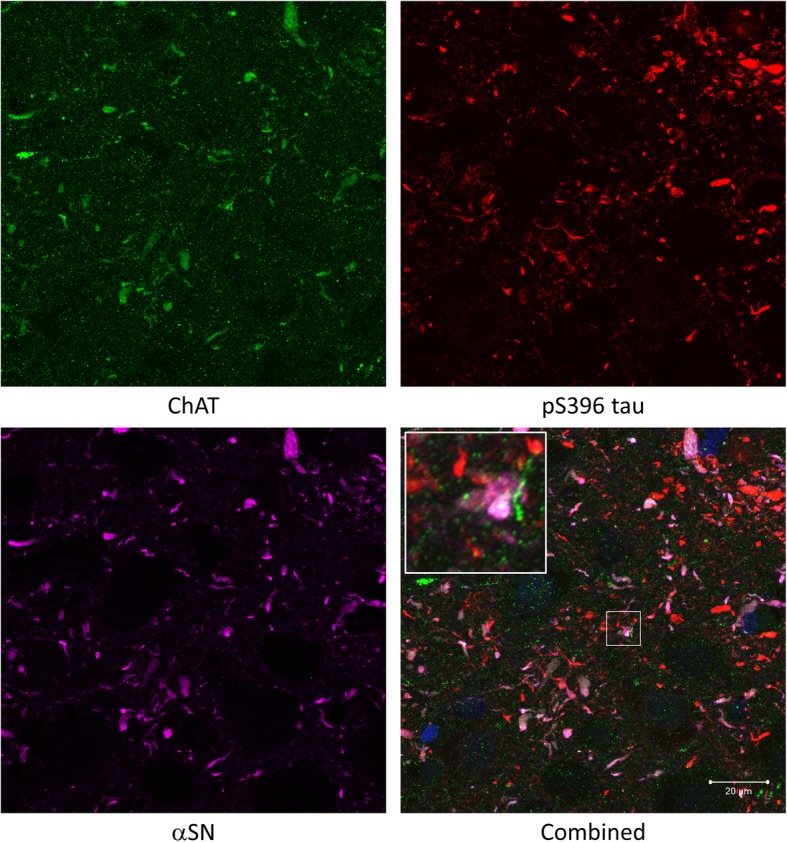
Triple immunofluorescence staining between ChAT (green), αSN (magenta) and pS396 tau (red). DAPI (blue) was also shown in combined image. A high degree of colocalisation between ChAT and αSN was observed and pS396 tau did not seem to colocalise with ChAT or αSN. Insets showing co-localisation in higher magnification. Scale bar = 20 μm

To investigate the relationship between cholinergic fibres and aggregated protein pathology in the CA2 hippocampal subfield, hippocampal sections of 1 PD and 1 PDD case with high tau and αSN burden and a long disease duration (19 and 20 years, respectively) were double or triple immunostained for ChAT, αSN and tau. A strong relationship between αSN and ChAT was found with double immunofluorescence (Fig. 6). Nearly all αSN was found to colocalise with ChAT, while not all ChAT fibres colocalise with αSN. Neuritic processes demonstrating colocalised with ChAT and αSN had morphology comparable to Lewy neurites. in areas where the load of αSN was low, the morphology of ChAT-positive fibres was not altered. In contrast, double immunofluorescence for ChAT and tau showed minimal or no colocalisation within the hippocampal CA2 subfield. It was observed that tau neuropil threads are wrapped around ChAT-positive fibres but no direct overlap between the two was found. Subsequently triple immunofluorescence staining further demonstrated large ChAT-positive neurites almost exclusively colocalised with αSN but not tau (Fig. 7). Colocalisation between all three staining was also observed, suggesting there may be a synergistic relationship between different pathogenic proteins.

## Discussion

This study supports the hypothesis that pathology within the hippocampal CA2 subfield contributes to cognitive decline in pure PD cases with no or minimal co-existing Alzheimer’s pathology. In particular, a high burden of Lewy pathology differentiated cases with dementia from those without. In addition, the loss of CA2 cholinergic fibres appeared to be a more sensitive marker as it distinguished PD cases with cognitive impairment from cases with no reported cognitive deficit. The significant association of a high density of hippocampal CA2 Lewy pathology with dementia in PD was consistent with previous observations [33, 47]. In a post-mortem study by Churchyard and Lees, significantly higher densities of Lewy pathology were only found in PD cases with severe dementia but not mild to moderate dementia [17]. Similarly, in the current study, we found that only in cases with dementia but not those with PD-MCI had a significantly higher degree of Lewy neurite burden, suggesting the deposition of Lewy pathology in the CA2 hippocampal subfield may be the end-stage process in cognitive decline in PD. However, it is important to note that even cases with no cognitive impairment can have some degree of αSN deposition. Hence, CA2 Lewy pathology alone is not sufficient in the diagnosis of PDD. Similar to the findings in DLB cases [22], we found no significant association between neuritic tau burden and dementia in PD, supporting the hypothesis that PDD and DLB lie in the same disease spectrum [31]. Although our group previously reported significant association between CA2 tau burden and PDD [47], the current study only showed a trend increase in tau pathology in PDD cases. This may be due to the difference in the assessment of tau pathology as the current study used neuritic tau burden rather than overall tau burden (neurofibrillary tangle + neurites) following the currently recommended criteria for the assessment of tau pathology outlined by BrainNet Europe [3]. In addition, the criteria for the neuropathological diagnosis of AD have been revised in recent years, meaning the cases selected from the earlier study may have a higher degree of co-existing AD pathology which would have been excluded in the current study.

The presence of hippocampal dopaminergic innervation has been extensively reported in rodents [60]. However, monoaminergic fibres were reported to be absent in the human hippocampal CA2/3 subfields using immunostaining with anti-tyrosine hydroxylase antibodies in DLB cases and controls [22]. By contrast, septohippocampal cholinergic innervation appeared to be preserved in humans as ChAT-immunopositive fibres and acetylcholinesterase staining were found in the hippocampus of aged brains, with highest density in the CA2 subfield [42, 64]. Using acetylcholinesterase histochemistry, it was previously reported that AD cases had a reduction in staining throughout the hippocampus with varying levels of enhancement found in different layers in the hippocampus, suggestive of sprouting of acetylcholinesterase terminals following neurodegeneration in the adjacent entorhinal cortex [42]. For PD, a significant degree of cholinergic depletion in the hippocampus was found in PDD cases when compared with PD and controls using ChAT activity radio-enzymatic assay on hippocampal brain homogenates [33]. However, specific changes in hippocampal cholinergic innervation were not studied. In the present study, we focussed on the CA2 hippocampal subfield and found significant cholinergic depletion not limited to PDD but also PD-MCI cases, when compared with PD cases without reported cognitive impairment. This suggests that there is a failure of cholinergic activity in advance of αSN deposition in the CA2 region, leading to the clinical progression to dementia. This is supported by pharmacological studies showing an improvement of objective cognitive measures in PD patients with cognitive impairment on cholinesterase inhibitors, irrespective of whether or not they have a diagnosis of dementia [1, 51]. Interestingly, we observed that there was great variation in cholinergic fibre densities within PD cases with no cognitive impairment, with some having low densities matching that of PDD and PD-MCI cases. This observed variability suggests that there may be compensatory aborisation of surviving cholinergic neurons, at least in a subset of PD cases without cognitive deficits. It can also be explained by the heterogeneity observed in clinical phenotypes, with previous studies reporting non-tremor dominant PD subtype or those with more prominent axial symptoms are more prone to progression to dementia [28, 65]. In future studies, it will be important to investigate if differing PD subtypes have varying baselines of cholinergic supply leading to an earlier or later presentation of cognitive symptoms.

Recent advancement in neuroscience techniques has led to better understanding of the precise function and connectivity of various hippocampal subfields. In particular, there has been a resurgence of interest in the CA2 region as its molecular signature was recently established in detail, distinguishing itself from the neighbouring CA1 and CA3 subfields [16, 24]. Selective vulnerability of hippocampal subfields has been reported in many neurological disorders. Neuronal loss were reported in the CA1 in AD [13]; CA2 in schizophrenia, bipolar disorder [11] and CA3 in stress-related conditions such as post-traumatic stress disorder, recurrent depression and Cushing’s syndrome [57].

In neurodegenerative conditions, specific subfield predilections have also been seen in aggregated protein pathology [7]. In AD, neurofibrillary tangles were preferentially found in the CA1 subfield [41] and they were found to correlate with neuronal loss and severity of cognitive impairment [13]. In DLB cases, Lewy pathologies were highest in CA2 and the entorhinal region. However, only CA1 burden correlates with cognitive decline using formal cognitive assessment [2]. In addition, it appeared that CA1 pathology could only be found in cases with pathology in the CA2 subfield, suggesting a possible hierarchical pattern of Lewy pathology spread in the hippocampus [2]. Hippocampal CA1 subfield is important for memory formation as specific bilateral CA1 lesion produced an amnesic syndrome [68]. Although memory deficit features in both DLB and AD, detectable memory impairment is normally apparent at a later stage in DLB [58], consistent with findings in previous studies. Similar to DLB, the CA2 subfield was also most affected in PDD cases with the greatest density of Lewy body, Lewy neurite and Lewy grain pathologies compared with other hippocampal and cortical regions [8]. Memory complaints were present in two-third of all PDD patients, but typically retrieval rather than storage and encoding memory (as seen in AD) was more affected. Also, in PDD, memory typically improves with cueing [27]. Owing to the specificity of αSN pathology deposition in Lewy body disease, it could be speculated that the CA2 subfield contributes to retrieval memory function to a certain extent. A recent study that investigated the different amnestic profiles between AD and PDD patients found free and cued memory recall was significantly impaired in both dementia groups but only PDD improves with total recall (with cueing). The authors attributed the difference in cognitive performance to structural and connectivity changes seen with magnetic resonance imaging (MRI) and diffuse tensor imaging (DTI) in CA1, CA4-DG and subiculum hippocampal subfields in AD, and the CA2–3 and presubiculum regions in PDD [61]. Similar observations were reported in an 18-month longitudinal MRI study on PD patients, which CA2–3 atrophy was found in patients converted to PDD compared with PD patients that remained cognitively unaffected [29]. These findings mirror the significant pathology we observed in the CA2 region of PD cases with cognitive impairment.

The connectivity of CA2 has been well described in rodents. Pyramidal neurons within the CA2 are the major output neurons to the CA1 and CA3 and they receive excitatory glutamatergic input from dentate gyrus and entorhinal cortex. They also receive input from other subcortical regions including the medial septum/ diagonal band, amygdala and hypothalamic nuclei. Thus, the CA2 was thought to be the integrating hub for cognitive and emotional functions [16]. In fact, rodent studies demonstrated the CA2’s role in social recognition memory and spatial processing [24], domains not formally assessed with standard cognitive assessments. The mechanism behind this remained to be discovered, but one study found that CA2 activity pattern changes over time despite surrounding spatial environment remains the same [55]. This suggests that CA2 may have important role in episodic memory, which is required for social memory formation. Whilst mood disturbance can be explained by CA2 neuronal loss in schizophrenia and bipolar cases [11], significant hippocampal subfield atrophy was not found in pure PD or DLB cases [36]. From our preliminary observations, it appeared that Lewy pathology in the CA2 exclusively affects cholinergic fibres in PD cases as found in the current study. Previously, the presence of acetylcholinesterase-positive neuritic plaque was found in the subiculum-CA1 area in AD cases [42]. In PD, colocalisation of Lewy bodies and ChAT-immunopositive neurons or neurites were found in the nbM and the pedunculopontine nucleus (PPN) and amygdala [25]. LB-infested neurons had a reduction of ChAT immunoreactivitiy, leading to the speculation that Lewy pathologies may disrupt cholinergic neurotransmission by the sequestration of ChAT enzyme [25]. To our knowledge, this is the first study to report specific colocalisation of Lewy neuritic pathology with ChAT-immunopositive fibres within the CA2 hippocampal subfield, although further studies using other antibodies that recognise misfolded αSN will be required to confirm the colocalisation of pathological αSN in cholinergic terminals. We therefore hypothesise that Lewy pathology-related cholinergic degeneration can lead to cognitive impairment in PD cases, through mechanisms as suggested above that pathology in the CA2 subfield may contribute to retrieval memory deficits in PD patients. In addition, we found CA2 cholinergic fibre depletion significantly correlated with the degeneration of cholinergic neurons in the nvlDBB. Although not established in primates, anterograde and retrograde tracer experiments using modified viral vector in mice has identified reciprocal connections between the medial septal nucleus-nvlDBB and CA2 [19]. A prion-like spread of αSN aggregation has been proposed to happen in interconnected regions [14]. Collectively, this supports the role of a hypothetical connection between CA2 and Ch2, where CA2-subregional Lewy pathology could have led to retrograde neurodegeneration of the cholinergic cells in nvlDBB, leading to dementia in PD.

## References

[1] Aarsland D, Laake K, Larsen JP, Janvin C (2002). Donepezil for cognitive impairment in Parkinson’s disease: a randomised controlled study. J Neurol Neurosurg Psychiatry.

[2] Adamowicz DH, Roy S, Salmon DP, Galasko DR, Hansen LA, Masliah E, Gage FH (2017). Hippocampal α-Synuclein in dementia with Lewy bodies contributes to memory impairment and is consistent with spread of pathology. J Neurosci.

[3] Alafuzoff I, Arzberger T, Al-Sarraj S, Bodi I, Bogdanovic N, Braak H, Bugiani O, Del-Tredici K, Ferrer I, Gelpi E, Giaccone G, Graeber MB, Ince P, Kamphorst W, King A, Korkolopoulou P, Kovács GG, Larionov S, Meyronet D, Monoranu C, Parchi P, Patsouris E, Roggendorf W, Seilhean D, Tagliavini F, Stadelmann C, Streichenberger N, Thal DR, Wharton SB, Kretzschmar H (2008). Staging of neurofibrillary pathology in Alzheimer’s disease: a study of the BrainNet Europe Consortium. Brain Pathol.

[4] Alafuzoff I, Ince PG, Arzberger T, Al-Sarraj S, Bell J, Bodi I, Bogdanovic N, Bugiani O, Ferrer I, Gelpi E, Gentleman S, Giaccone G, Ironside JW, Kavantzas N, King A, Korkolopoulou P, Kovács GG, Meyronet D, Monoranu C, Parchi P, Parkkinen L, Patsouris E, Roggendorf W, Rozemuller A, Stadelmann-Nessler C, Streichenberger N, Thal DR, Kretzschmar H (2009). Staging/typing of Lewy body related alpha-synuclein pathology: a study of the BrainNet Europe consortium. Acta Neuropathol.

[5] American Psychiatric Association (2000) Diagnostic and Statistical Manual of Mental Disorders, Fourth Edition, Text Revision (DSM-IV-TR) [Internet]. American Psychiatric Association

[6] Arellano JI, Muñoz A, Ballesteros-Yáñez I, Sola RG, DeFelipe J (2004). Histopathology and reorganization of chandelier cells in the human epileptic sclerotic hippocampus. Brain.

[7] Armstrong RA, Cairns NJ (2015). Comparative quantitative study of “signature” pathological lesions in the hippocampus and adjacent gyri of 12 neurodegenerative disorders. J Neural Transm Springer Vienna.

[8] Armstrong RA, Kotzbauer PT, Perlmutter JS, Campbell MC, Hurth KM, Schmidt RE, Cairns NJ (2014). A quantitative study of α-synuclein pathology in fifteen cases of dementia associated with Parkinson disease. J Neural Transm.

[9] Ballard C, Ziabreva I, Perry R, Larsen JP, O’Brien J, McKeith I, Perry E, Aarsland D (2006). Differences in neuropathologic characteristics across the Lewy body dementia spectrum. Neurology.

[10] Bartus RT, Dean RL, Beer B, Lippa A (1982). The cholinergic hypothesis of geriatric memory dysfunction. Science..

[11] Benes FM, Kwok EW, Vincent SL, Todtenkopf MS (1998). A reduction of nonpyramidal cells in sector CA2 of schizophrenics and manic depressives. Biol Psychiatry.

[12] Blümcke I, Thom M, Aronica E, Armstrong DD, Bartolomei F, Bernasconi A, Bernasconi N, Bien CG, Cendes F, Coras R, Cross JH, Jacques TS, Kahane P, Mathern GW, Miyata H, Moshé SL, Oz B, Özkara Ç, Perucca E, Sisodiya S, Wiebe S, Spreafico R (2013). International consensus classification of hippocampal sclerosis in temporal lobe epilepsy: a task force report from the ILAE commission on diagnostic methods. Epilepsia.

[13] Bobinski M, Wegiel J, Tarnawski M, Bobinski M, Reisberg B, de Leon MJ, Miller DC, Wisniewski HM (1997). Relationships between regional neuronal loss and neurofibrillary changes in the hippocampal formation and duration and severity of Alzheimer disease. J Neuropathol Exp Neurol.

[14] Brundin P, Melki R, Kopito R (2010). Prion-like transmission of protein aggregates in neurodegenerative diseases. Nat Rev Mol Cell Biol.

[15] Chaudhuri KR, Healy DG, Schapira AHV (2006). National Institute for clinical excellence. Non-motor symptoms of Parkinson’s disease: diagnosis and management. Lancet Neurol.

[16] Chevaleyre V, Piskorowski RA (2016). Hippocampal area CA2: an overlooked but promising therapeutic target. Trends Mol Med Elsevier Ltd.

[17] Churchyard A, Lees A (1997). The relationship between dementia and direct involvement of the hippocampus and amygdala in Parkinson’s disease. Neurology.

[18] Compta Y, Parkkinen L, O’Sullivan SS, Vandrovcova J, Holton JL, Collins C, Lashley T, Kallis C, Williams DR, de Silva R, Lees AJ, Revesz T (2011). Lewy- and Alzheimer-type pathologies in Parkinson’s disease dementia: which is more important?. Brain.

[19] Cui Z, Gerfen CR, Young WS (2013). Hypothalamic and other connections with dorsal CA2 area of the mouse hippocampus. J Comp Neurol.

[20] Daniel SE, Lees AJ (1993). Parkinson’s disease society brain Bank, London: overview and research. J Neural Transm Suppl.

[21] Dickson DW, Ruan D, Crystal H, Mark MH, Davies P, Kress Y, Yen SH (1991). Hippocampal degeneration differentiates diffuse Lewy body disease (DLBD) from Alzheimer’s disease: light and electron microscopic immunocytochemistry of CA2-3 neurites specific to DLBD. Neurology AAN Enterprises.

[22] Dickson DW, Schmidt ML, Lee VM, Zhao ML, Yen SH, Trojanowski JQ (1994). Immunoreactivity profile of hippocampal CA2/3 neurites in diffuse Lewy body disease. Acta Neuropathol.

[23] Docherty MJ, Burn DJ (2010). Parkinson’s disease dementia. Curr Neurol Neurosci Rep.

[24] Dudek SM, Alexander GM, Farris S (2016) Rediscovering area CA2: unique properties and functions. Nat Rev Neurosci 17(2):89–102. 10.1038/nrn.2015.22 PMC4856153 26806628

[25] Dugger BN, Dickson DW (2010). Cell type specific sequestration of choline acetyltransferase and tyrosine hydroxylase within Lewy bodies. Acta Neuropathol.

[26] Emre M (2003). Dementia associated with Parkinson’s disease. Lancet Neurol.

[27] Emre M, Aarsland D, Brown R, Burn DJ, Duyckaerts C, Mizuno Y, Broe GA, Cummings J, Dickson DW, Gauthier S, Goldman J, Goetz C, Korczyn A, Lees A, Levy R, Litvan I, McKeith I, Olanow W, Poewe W, Quinn N, Sampaio C, Tolosa E, Dubois B (2007). Clinical diagnostic criteria for dementia associated with Parkinson’s disease. Mov Disord.

[28] Evans JR, Mason SL, Williams-Gray CH, Foltynie T, Brayne C, Robbins TW, Barker RA (2011). The natural history of treated Parkinson’s disease in an incident, community based cohort. J Neurol Neurosurg Psychiatry.

[29] Foo H, Mak E, Chander RJ, Ng A, Au WL, Sitoh YY, Tan LCS, Kandiah N (2017). Associations of hippocampal subfields in the progression of cognitive decline related to Parkinson’s disease. NeuroImage Clin.

[30] Fujishiro H, Umegaki H, Isojima D, Akatsu H, Iguchi A, Kosaka K (2006). Depletion of cholinergic neurons in the nucleus of the medial septum and the vertical limb of the diagonal band in dementia with Lewy bodies. Acta Neuropathol.

[31] Goldman JG, Williams-Gray C, Barker RA, Duda JE, Galvin JE (2014). The spectrum of cognitive impairment in Lewy body diseases. Mov Disord.

[32] Halgin R, Riklan M, Misiak H (1977). Levodopa, parkinsonism, and recent memory. J Nerv Ment Dis.

[33] Hall H, Reyes S, Landeck N, Bye C, Leanza G, Double K, Thompson L, Halliday G, Kirik D (2014) Hippocampal Lewy pathology and cholinergic dysfunction are associated with dementia in Parkinson’s disease. Brain 137(9):2493-2508 10.1093/brain/awu193 25062696

[34] Halliday G, Hely M, Reid W, Morris J (2008). The progression of pathology in longitudinally followed patients with Parkinson’s disease. Acta Neuropathol.

[35] Halliday GM, Cullen K, Cairns MJ (1993). Quantitation and three-dimensional reconstruction of Ch4 nucleus in the human basal forebrain. Synapse.

[36] Harding AJ, Lakay B, Halliday GM (2002). Selective hippocampal neuron loss in dementia with Lewy bodies. Ann Neurol.

[37] Hely MA, Reid WGJ, Adena MA, Halliday GM, Morris JGL (2008). The Sydney multicenter study of Parkinson’s disease: the inevitability of dementia at 20 years. Mov Disord.

[38] Hietanen M, Teräväinen H (1988). Dementia and treatment with L-dopa in Parkinson’s disease. Mov Disord.

[39] Horvath J, Herrmann FR, Burkhard PR, Bouras C, Kövari E (2013). Neuropathology of dementia in a large cohort of patients with Parkinson’s disease. Parkinsonism Relat Disord Elsevier Ltd.

[40] Howlett DR, Whitfield D, Johnson M, Attems J, O’Brien JT, Aarsland D, Lai MKP, Lee JH, Chen C, Ballard C, Hortobágyi T, Francis PT (2015). Regional multiple pathology scores are associated with cognitive decline in Lewy body dementias. Brain Pathol.

[41] Hyman B, Van Hoesen G, Damasio A, Barnes C (1984). Alzheimer’s disease: cell-specific pathology isolates the hippocampal formation. Science (80- ).

[42] Hyman BT, Kromer LJ, Van Hoesen GW (1987). Reinnervation of the hippocampal perforant pathway zone in Alzheimer’s disease. Ann Neurol.

[43] Ince PG, Holton JL, Revesz T, Wharton SB (2008) Diseases of movement and system degenerations. In: Love S, Louis DN, Ellison DW (eds) Greenfield’s neuropathology, 8th edn. Hodder Arnold, pp 889–1030

[44] Irwin DJ, White MT, Toledo JB, Xie SX, Robinson JL, Van Deerlin V, Lee VM-Y, Leverenz JB, Montine TJ, Duda JE, Hurtig HI, Trojanowski JQ (2012). Neuropathologic substrates of Parkinson disease dementia. Ann Neurol.

[45] Jellinger KA, Attems J (2008). Prevalence and impact of vascular and Alzheimer pathologies in Lewy body disease. Acta Neuropathol.

[46] Kai H, Shin R-W, Ogino K, Hatsuta H, Murayama S, Kitamoto T (2012). Enhanced antigen retrieval of amyloid β immunohistochemistry: re-evaluation of amyloid β pathology in Alzheimer disease and its mouse model. J Histochem Cytochem.

[47] Kalaitzakis ME, Christian LM, Moran LB, Graeber MB, Pearce RKB, Gentleman SM (2009). Dementia and visual hallucinations associated with limbic pathology in Parkinson’s disease. Parkinsonism Relat Disord. Elsevier Ltd.

[48] Kalaitzakis ME, Pearce RKB (2009). The morbid anatomy of dementia in Parkinson’s disease. Acta Neuropathol.

[49] Kövari E, Gold G, Herrmann FR, Canuto A, Hof PR, Bouras C, Giannakopoulos P (2003). Lewy body densities in the entorhinal and anterior cingulate cortex predict cognitive deficits in Parkinson’s disease. Acta Neuropathol.

[50] Lees AJ, Hardy J, Revesz T (2009). Parkinson’s disease. Lancet Elsevier Ltd.

[51] Leroi I, Brandt J, Reich SG, Lyketsos CG, Grill S, Thompson R, Marsh L (2004). Randomized placebo-controlled trial of donepezil in cognitive impairment in Parkinson’s disease. Int J Geriatr Psychiatry.

[52] Lippa CF, Duda JE, Grossman M, Hurtig HI, Aarsland D, Boeve BF, Brooks DJ, Dickson DW, Dubois B, Emre M, Fahn S, Farmer JM, Galasko D, Galvin JE, Goetz CG, Growdon JH, Gwinn-Hardy KA, Hardy J, Heutink P, Iwatsubo T, Kosaka K, Lee VM-Y, Leverenz JB, Masliah E, IG MK, Nussbaum RL, Olanow CW, Ravina BM, Singleton AB, Tanner CM, Trojanowski JQ, Wszolek ZK, DLB/PDD Working Group (2007). DLB and PDD boundary issues: diagnosis, treatment, molecular pathology, and biomarkers. Neurology.

[53] Litvan I, Goldman JG, Tröster AI, Schmand BA, Weintraub D, Petersen RC, Mollenhauer B, Adler CH, Marder K, Williams-Gray CH, Aarsland D, Kulisevsky J, Rodriguez-Oroz MC, Burn DJ, Barker RA, Emre M (2012). Diagnostic criteria for mild cognitive impairment in Parkinson’s disease: Movement Disorder Society task force guidelines. Mov Disord.

[54] Liu AKL, Lim EJ, Ahmed I, Chang RC-C, Pearce RKB, Gentleman SM (2018). Review: revisiting the human cholinergic nucleus of the diagonal band of Broca. Neuropathol Appl Neurobiol.

[55] Mankin EA, Diehl GW, Sparks FT, Leutgeb S, Leutgeb JK (2015). Hippocampal CA2 activity patterns change over time to a larger extent than between spatial contexts. Neuron.

[56] Mattila PM, Rinne JO, Helenius H, Dickson DW, Röyttä M (2000). Alpha-synuclein-immunoreactive cortical Lewy bodies are associated with cognitive impairment in Parkinson’s disease. Acta Neuropathol.

[57] McEwen BS (1997). Magarinos a M. stress effects on morphology and function of the hippocampus. Ann N Y Acad Sci.

[58] McKeith IG, Boeve BF, Dickson DW, Halliday G, Taylor J-P, Weintraub D, Aarsland D, Galvin J, Kosaka K (2017). Diagnosis and management of dementia with Lewy bodies: fourth consensus report of the DLB consortium. Neurology..

[59] Mesulam MM, Mufson EJ, Levey AI, Wainer BH (1983). Cholinergic innervation of cortex by the basal forebrain: cytochemistry and cortical connections of the septal area, diagonal band nuclei, nucleus basalis (substantia innominata), and hypothalamus in the rhesus monkey. J Comp Neurol.

[60] Milner TA, Bacon CE (1989). Ultrastructural localization of tyrosine hydroxylase-like immunoreactivity in the rat hippocampal formation. J Comp Neurol.

[61] Novellino F, Vasta R, Alessia S, Chiriaco C, Maria S, Maurizio M, Gennarina A, Saccà V, Nicoletti G, Quattrone A (2017) Relationship between hippocampal subfields and category cued recall in AD and PDD: a multimodal MRI study. Neuroscience. 10.1016/j.neuroscience.2017.12.028 29292073

[62] Pang CC-C, Kiecker C, O’Brien JT, Noble W, Chang RC-C (2018) Ammon’s horn 2 (CA2) of the Hippocampus: a long-known region with a new potential role in neurodegeneration. Neuroscientist :1073858418778747 10.1177/1073858418778747 29865938

[63] Perry EK, Curtis M, Dick DJ, Candy JM, Atack JR, Bloxham CA, Blessed G, Fairbairn A, Tomlinson BE, Perry RH (1985). Cholinergic correlates of cognitive impairment in Parkinson’s disease: comparisons with Alzheimer’s disease. J Neurol Neurosurg Psychiatry.

[64] Ransmayr G, Cervera P, Hirsch E, Ruberg M, Hersh LB, Duyckaerts C, Hauw J-J, Delumeau C, Agid Y (1989). Choline acetyltransferase-like immunoreactivity in the hippocampal formation of control subjects and patients with Alzheimer’s disease. Neuroscience..

[65] Selikhova M, Williams DR, Kempster PA, Holton JL, Revesz T, Lees AJ (2009). A clinico-pathological study of subtypes in Parkinson’s disease. Brain.

[66] Tsuboi Y, Dickson DW (2005). Dementia with Lewy bodies and Parkinson’s disease with dementia: are they different?. Parkinsonism Relat Disord.

[67] World Health Organization (1992). The ICD-10 classification of mental and behavioural disorders: clinical descriptions and diagnostic guidelines.

[68] Zola-Morgan S, Squire LR, Amaral DG (1986). Human amnesia and the medial temporal region: enduring memory impairment following a bilateral lesion limited to field CA1 of the hippocampus. J Neurosci.

